# Childhood Trauma is Associated with Altered Cortical Arousal: Insights from an EEG Study

**DOI:** 10.3389/fnint.2012.00120

**Published:** 2012-12-24

**Authors:** Fleur Margaret Howells, Dan J. Stein, Vivienne A. Russell

**Affiliations:** ^1^Department of Human Biology, Faculty of Health Sciences, University of Cape TownCape Town, South Africa; ^2^Department of Psychiatry, Faculty of Health Sciences, University of Cape TownCape Town, South Africa

**Keywords:** physical neglect, physical abuse, emotional neglect, emotional abuse, sexual abuse, denial

## Abstract

**Background:** Childhood trauma is associated with psychiatric disorders, yet the underlying psychobiological mechanisms that account for this link are not well understood. Alterations in cortical arousal may, however, play a key role in mediating this association. We hypothesized that childhood trauma would be associated with alterations in arousal during a task that required sustained attention and behavioral inhibition. **Materials and Methods:** Fifty-three healthy adults completed the Childhood Trauma Questionnaire which assesses physical neglect, emotional neglect, emotional abuse, physical abuse, sexual abuse, and denial of childhood trauma. These individuals underwent cortical (electroencephalography) and peripheral (heart rate, skin conductance responses, and salivary cortisol) physiological recordings at rest (eyes open and eyes closed) and during performance of a visual go/no-go (GNG) task. Associations between reported childhood trauma and physiological measures were determined. **Results:** Physical and emotional neglect were correlated with decreased left parietal tonic α band power during resting conditions and during the GNG task. Emotional abuse was correlated with decreased right frontal α band power during rest, increased θ band power during the GNG task, and cortisol at the end of the testing session. Physical and sexual abuse were correlated with delayed P300 latency and enhanced P300 amplitude during the no-go conditions of the GNG task. The denial scale was correlated with a decrease in θ and increase in α band power during the no-go conditions of the GNG task. **Conclusion:** The present study provides evidence that childhood trauma is associated with altered cortical arousal and that the pattern of this association is dependent on the form of childhood trauma experienced.

## Introduction

A significant association between childhood trauma and subsequent psychiatric disorder has been well documented (Briere and Runtz, 1988; Hirschfield et al., 1991; Millon, 1991; de Wilde et al., 1992; Gross and Keller, 1992; Kendler et al., 1995; Bernstein et al., 1998; Pynoos et al., 1999; Kendall-Tackett, 2000; Dube et al., 2001, 2003; Johnson et al., 2002; De Sanctis et al., 2008). Nevertheless, the underlying psychobiological mechanisms that account for this link are not well understood. However, as the association between childhood trauma and psychopathology is not restricted to any particular psychiatric disorder, it is likely that such mechanisms involve quite general aspects of mental processing. As both hypo-arousal and hyper-arousal are associated with suboptimal responses to internal and external stimuli, and so impact negatively on behavioral performance, one possibility is that alterations in arousal play a key role in mediating the relationship between early trauma and subsequent disorder (Howells et al., 2012).

Key arousal systems of the central nervous system include: (1) the locus coeruleus norepinephrine (LC-NE) system; (2) magnocellular basal forebrain/pedunculopontine cholinergic system; (3) substantia nigra/ventral tegmental area dopaminergic system; (4) dorsal raphe serotonergic system; and (5) tuberomamillary hypothalamic histaminergic system (Marrocco et al., 1994). Cortical arousal as measured by electroencephalography (EEG) provides an important window on these different systems. Peripheral arousal is strongly related to activity of the sympathetic and parasympathetic nervous system, and can be assessed through measurement of heart rate, skin conductance, and cortisol (Kendall-Tackett, 2000; Heim and Nemeroff, 2001). Cortical arousal can be divided into tonic and phasic components. Tonic cortical arousal is determined by continuous EEG recordings from which frequency band power, such as theta (θ, 4–7 Hz), alpha (α, 7–14 Hz), and beta (β, 15–30 Hz) band power, can be derived. Decreased tonic cortical arousal has been related to increased theta band power (Lansbergen et al., 2007; Barry et al., 2009; VaezMousavi et al., 2009) and decreased beta band power (Lansbergen et al., 2007), while increased tonic cortical arousal is reflected in increased beta band power (Sachs et al., 2004; Grin-Yatsenko et al., 2009, 2010). A higher ratio of theta/beta EEG frequency band power is frequently used to reflect decreased tonic arousal, which has been associated with poor attentional function (Mann et al., 1992) and reduced activation of neural circuitry (Polich and Kok, 1995; Lazzaro et al., 1999; Barry et al., 2009). If the tonic level of arousal is not optimal and the individual is required to complete a cognitive task, phasic arousal will be affected (Sokolov, 1960; Donchin and Coles, 1988; Polich and Kok, 1995; Howells et al., 2012). This will be evidenced by impaired performance and by attenuation of and/or delay in cortical event-related potential (ERP) components during the task (Sokolov, 1960; Donchin and Coles, 1988; Polich and Kok, 1995; Howells et al., 2012).

A cognitive task, such as the go/no-go (GNG) task, which requires sustained attention and behavioral inhibition, may be particularly relevant to the assessment of altered arousal in individuals with a history of childhood trauma (Bokura et al., 2001; Posner and Rothbart, 2007). To sustain attention the individual is required to activate three networks: alerting, orientating, and executive (Posner and Rothbart, 2007). The alerting network is involved in the acquisition and maintenance of the alert state required to perform the task. The orienting network directs the individual to the stimuli that need to be attended to. The executive network facilitates the resolution of conflict between performance and affect, and so mediates behavioral inhibition (Posner and Petersen, 1990; Coull et al., 1996; Coull, 1998; Posner et al., 2006). Previous work has established that behavioral inhibition is an essential regulatory function that develops progressively from childhood to adulthood (Williams et al., 1999), and that is impaired in those with a history of childhood trauma (Anda et al., 2006; Hart and Rubia, 2012).

We explore the possibility that childhood trauma experienced by healthy individuals would be associated with altered arousal. These changes in arousal would be dependent on the interaction between the individuals’ tonic arousal and phasic arousal. Physiological measures of arousal were recorded during resting conditions and during several conditions of a visual GNG task, a task that requires sustained attention and behavioral inhibition.

## Materials and Methods

### Participants

Fifty-three participants (22 males, 31 females, 27 ± 0.8 years old) were recruited from the postgraduate community of the University of Cape Town, South Africa. This cohort included personnel, ranging from students through to postdoctoral fellows and administrators. Participation held no incentive, was voluntary, and the participants knew the study was for research purposes only. The study was approved by the Faculty of Health Sciences Human Ethics Committee of the University of Cape Town, and the participants signed informed consent. The study was conducted in accordance with the Declaration of Helsinki (WMA General Assembly, 2000). Participants reported no psychiatric or substance use disorders, no use of any psychoactive medications, and did not have a current general medical condition or a history of brain trauma. Participants were proficient in English, having been taught in English during their schooling.

### Experimental design

Participants were required to refrain from caffeine, cigarettes, alcohol, and non-prescription drugs for a minimum of 18 h prior to their testing session and were not in a state of distress – that is, did not report significant levels of physical training or environmental stressors. Physiological parameters were recorded between 09h30 and 13h30 using a MP150 Biopac acquisition system and Acqknowledge 3.8.1software (Biopac Systems Inc.) with amplifier modules for EEG, skin conductance response, and electrocardiograph. The testing session included three stages: (1) resting eyes open (REO, 2 min); (2) resting eyes closed (REC, 2 min); and (3) a visual letter GNG task. All stages of the testing session were programmed in Eprime 1.1, which sent digital inputs to physiological recording equipment. The testing session was completed in a quiet, unlit room to reduce distraction. Saliva samples were collected immediately before and after the testing session, to measure cortisol. All recordings and samples were taken between 09h30 and 13h30. All data analyses were performed after data acquisition, using the 3.8.1 Acqknowledge software.

### Measure of childhood trauma

Participants completed Bernstein’s Childhood Trauma Questionnaire – short form (Bernstein et al., 2003). This questionnaire included subscales of physical neglect, emotional neglect, emotional abuse, physical abuse, sexual abuse, and denial.

### Visual go/no-go task

The GNG required sustained attention: it had conditions that required inhibition and/or delayed stimulus presentation. The participant responded by button press to all consonants with the exception of the letter “V.” No vowels were used. There were five conditions in the GNG task, a total of 200 trials. The first condition of the GNG was a go condition, in which all stimuli required responses by the participant, with an inter-stimulus interval of 1500 ms. The second condition of the GNG was a no-go condition, in which participants were required to respond to all stimuli except the no-go stimulus, the letter V, with an inter-stimulus interval of 1500 ms. The third condition of the GNG was a go condition with an inter-stimulus interval of 3500 ms. The first and second conditions of the GNG were repeated in reverse after the third condition of the GNG. Each of the five conditions had 40 stimuli, in the no-go conditions 20 stimuli were go trials and 20 stimuli were no-go trials (50% split).

The GNG task is designed to include several aspects of executive function, voluntary sustained attention is required throughout the task, the go 1500 conditions (first and fifth condition) can be seen as a “control setting,” the no-go conditions activate response inhibition circuitry (second and fourth condition), and the go 3500 ms condition assesses the individual’s ability to delay their responding and maintain their attention on the task at hand (third condition). The design of the task permits a balance between these modes of cortical activity. The literature reports deficits in all three modalities of executive function in those that have experienced childhood trauma (Posner and Petersen, 1990; Coull et al., 1996; Coull, 1998; Bokura et al., 2001; Anda et al., 2006; Posner et al., 2006; Posner and Rothbart, 2007; Hart and Rubia, 2012). The GNG task and its conditions are therefore appropriate to interrogate the effects of childhood trauma on different brain circuitry that has been implicated in adults who have experienced childhood trauma.

### Cortical physiological measures (EEG) of arousal

The EEG data were collected with the use of EEG100C amplifier modules that were attached to the MP150 acquisition system (Biopac Systems Inc.). Electrodes of interest included: left frontal (F_3_), right frontal (F_4_), left parietal (P_3_), and right parietal (P_4_), with linked ears reference. The EEG data were sampled at 500 Hz on-line; band pass filtered using a Hamming window, 0.05–30 Hz off-line. Relative frequency band powers were extracted after the data had been subjected to Fast Fourier transformation. The relative frequency band powers extracted include: theta (θ, 4–7 Hz), alpha (α, 7–14 Hz), and beta (β, 15–30 Hz).

The ERPs were extracted from digital inputs (using E-prime software) for each of the GNG conditions. Individual ERPs that were greater than +100 μV or less than -100 μV were rejected. The ERPs were baseline corrected 100 ms prior to stimulus presentation, and visually inspected. The P300 window was set between 250 and 500 ms after stimulus presentation, the amplitude was the point at which the height of the P300 peak was maximal and latency the time taken to achieve maximum amplitude. The P300 amplitudes and latencies were extracted from the parietal electrodes (P_3_ and P_4_).

### Peripheral physiological measures of arousal

#### Skin conductance responses and heart rate

Skin conductance responses were recorded with the GSR100C amplifier module that was attached to the MP150 acquisition system (Biopac Systems Inc.). The GSR100C module was set to measure phasic activity (AC). Skin conductance responses were taken from distal phalanges of the non-dominant hand, with a sampling rate of 500 Hz on-line, the data were filtered using a Hamming window of 0.05–10 Hz off-line. The data were then analyzed for peaks exceeding a threshold value of 0.05 μϑ. The number of these peaks and the duration of these peaks were taken as the number of skin conductance responses and duration of skin conductance response (Boucsein, 1992).

Heart rate was determined from a five lead electrocardiogram recorded via ECG100C amplifier modules that were attached to the MP150 acquisition system (Biopac Systems Inc.). The data were collected at a sampling rate of 500 Hz on-line. The data were filtered using a Hamming window of 0.05–35 Hz off-line and heart rates were calculated using the 3.8.1 Acqknowledge software.

#### Salivary cortisol

Salivary cortisol was collected before and after the testing session with use of Salivettes^®^ (Sarstedt non-citric acid sterile cotton wool rolls). Samples were stored at -80°C. Salimetrics LLC expanded range high sensitivity salivary cortisol enzyme immunoassay kits were used to determine cortisol concentrations (Salimetrics Catalog No. 1-3002/1-3012, 96-Well Kit, lower detection limit 0.003 μg/dL, 0.083 nmol/L).

### Statistical analysis

Statistica 9 was used for the statistical analyses. Non-parametric statistics were used to analyze all data, since the Shapiro–Wilks *W* test revealed that the data set was not normally distributed.

To determine significant differences between stages (resting eyes open (REO), resting eyes closed (REC), and the GNG task) and conditions of the GNG task (first go condition, first no-go condition, go 3500 condition, second no-go condition, and second go condition) Friedman analyses of variance (ANOVA) were performed. If the ANOVA revealed a significant effect, it was followed by a Wilcoxon matched pairs test. Data are reported as mean ± SEM.

To determine significant correlations between the scores on the Childhood Trauma Questionnaire and physiological measurements recorded during the testing stages (resting eyes open, resting eyes closed, and the GNG task) and conditions of the GNG task (first go condition, first no-go condition, go 3500 condition, second no-go condition, and second go condition) Spearman’s rank order correlation analyses were performed. These data are reported as rho coefficients with their *p*-values.

## Results

### Childhood trauma questionnaire

The average rating of the 53 participants for the Childhood Trauma Questionnaire was 33.35 ± 1.04 without the denial scale and 34.11 ± 0.99 with the denial scale. The average ratings of the 53 participants on each subscale of the Childhood Trauma Questionnaire were as follows: physical abuse = 5.9 ± 0.17; physical neglect = 6.2 ± 0.25; emotional abuse = 7.6 ± 0.43; denial = 0.75 ± 0.15; emotional neglect = 8.3 ± 0.46; and sexual abuse = 5.2 ± 0.10. Overall the ratings given by the present cohort were lower than those reported in the normative data study conducted in American communities (Bernstein et al., 2003).

#### Cortical physiological measures (EEG) during the various stages of the testing session (REO, REC, and GNG)

**Figure 1:**
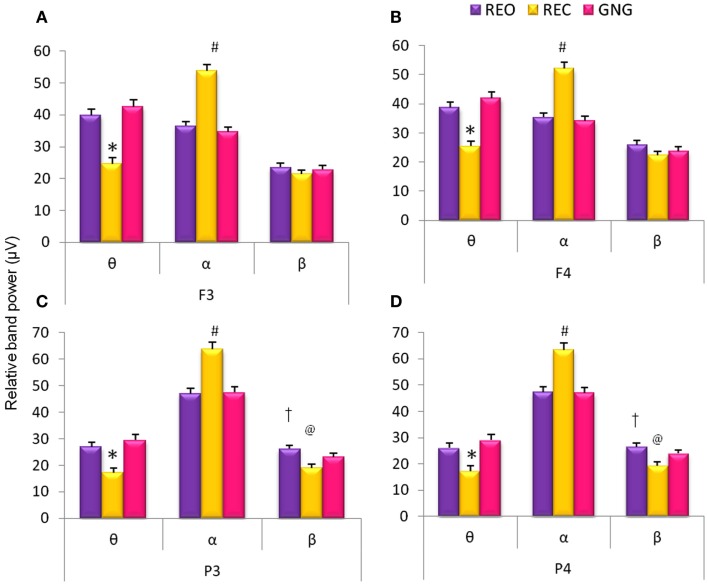
**Relative EEG frequency band power during different stages of the GNG testing session: resting eyes open (REO), resting eyes closed (REC), and the GNG task**. Relative frequencies reported: theta (θ, 4–7 Hz), alpha (α, 7–14 Hz), and beta (β, 15–30 Hz) for frontal (F_3_ and F_4_) and parietal (P_3_ and P_4_) electrodes. *For all electrodes relative θ band power was lower during REC compared to REO and GNG. ^#^For all **(A–D)** electrodes relative α band power was higher during REC than REO and GNG. ^@^Parietal electrodes **(C,D)** β band power was lower during REC than REO and GNG and ^†^higher during REO compared to GNG (*p* < 0.02, *n* = 53, mean ± SEM).

Comparison of relative EEG band power revealed significant differences between the various stages of the testing session (REO, REC, and GNG) in relative θ and α band power at frontal electrodes [F_3_θ χ^2^_(2,53)_ = 45.62, F_3_α χ^2^_(2,53)_ = 62.60, F_4_θ χ^2^_(2,53)_ = 52.98, F_4_α χ^2^_(2,53)_ = 57.39, *p* < 0.0001] and relative θ, α, and β band power at parietal electrodes [P_3_θ χ^2^_(2,53)_ = 45.32, P_3_α χ^2^_(2,53)_ = 53.09, P_3_β χ^2^_(2,53)_ = 25.92, P_4_θ χ^2^_(2,53)_ = 45.32, P_4_α χ^2^_(2,53)_ = 50.07, P_4_β χ^2^_(2,53)_ = 27.62, *p* < 0.0001]. Alpha band power was increased during REC compared with REO and the GNG task for frontal and parietal electrodes, as expected; that is, due to lack of visual sensory information and disengagement of thalamocortical networks. This was in turn reflected in the reduced relative θ band power during REC globally (frontal and parietal electrodes) and reduced β band power parietally (Figure 1).

**Figure 2:**
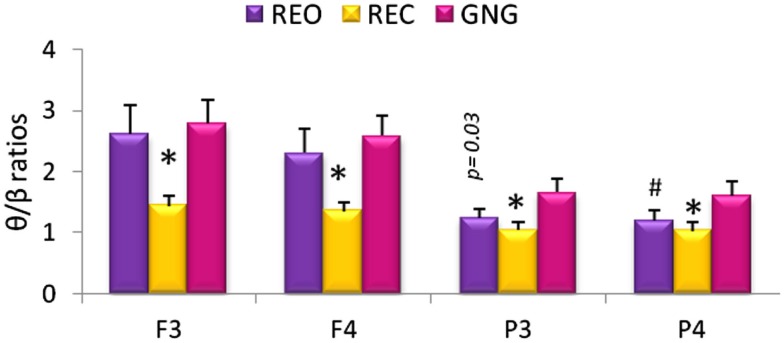
**Relative theta/beta (θ/β) ratios for frontal (F_3_ and F_4_) and parietal (P_3_ and P_4_) electrodes during different stages of the testing session: resting eyes open (REO), resting eyes closed (REC), and the GNG task**. *θ/β ratio was higher during REO and GNG compared to REC for all electrodes. ^#^Left parietal θ/β ratio was lower during REO compared to GNG θ/β ratio (*p* < 0.02 *n* = 53, mean ± SEM).

Differences between the various stages of the testing session (REO, REC, and GNG) were observed for θ/β ratios at all four electrodes [F_3_θ/β χ^2^_(2,53)_ = 29.92, F_4_θ/β χ^2^_(2,53)_ = 24.60, P_3_θ/β χ^2^_(2,53)_ = 17.09, P_4_θ/β χ^2^_(2,53)_ = 19.13, *p* < 0.0001]. Theta/beta ratios were increased globally during REO and GNG compared to REC, reflecting the disengagement of cortical networks related to changes in α band power. Right parietal (P_4_) θ/β was lower during REO compared to GNG, and a similar tendency (*p* = 0.03) was observed for left parietal (P_3_) θ/β ratios, reflecting increased cognitive activity during the GNG (Figure 2).

#### Associations between perceived childhood trauma and cortical physiological measures (EEG) during the various stages of the testing session (REO, REC, and GNG)

Childhood trauma scores correlated significantly with cortical measures of arousal during the various stages of the testing session (REO, REC, and GNG; Table 1). Similar correlates were found for the left hemisphere with and without inclusion of the denial scale. Childhood trauma (with and without denial scale) was significantly negatively correlated with left parietal (P_3_) α band power at all stages of the testing session, and with left parietal (P_3_) θ and β band power during REC. These correlates were stronger without inclusion of the ratings for the denial scale. Childhood trauma (with and without denial scale) was significantly negatively correlated with left frontal (F_3_) α band power during the REC stage and right frontal (F_4_) α band power during REO.

**Table 1:** **Correlations between the participants’ ratings, overall, and for the subscales, of the Childhood Trauma Questionnaire with cortical physiological EEG measures during the stages of the testing session**.

		CTQ score without denial scale						CTQ score with denial scale					
		Eyes open		Eyes closed		Go/no-go task		Eyes open		Eyes closed		Go/no-go task	
		Spearman	*p*-Value	Spearman	*p*-Value	Spearman	*p*-Value	Spearman	*p*-Value	Spearman	*p*-Value	Spearman	*p*-Value
**Left frontal (F_3_)**													
Relative θ (4–7 Hz) power		0.04	0.791	0.23	0.092	0.15	0.275	0.02	0.903	0.19	0.167	0.14	0.333
Relative α (7–13 Hz) power		−0.25	0.075	−0.32^#^	0.017	−0.18	0.190	−0.21	0.138	−0.27^#^	0.045	−0.15	0.286
Relative β (13–30 Hz) power		0.10	0.454	0.24	0.088	−0.05	0.717	0.10	0.493	0.16	0.246	−0.06	0.689
θ/β ratio		−0.03	0.828	0.02	0.898	0.10	0.461	−0.03	0.825	0.04	0.794	0.10	0.489
**Right frontal (F_4_)**													
Relative θ (4–7 Hz) power		0.09	0.529	0.19	0.163	0.19	0.164	0.10	0.459	0.17	0.232	0.19	0.169
Relative α (7–13 Hz) power		−0.30^#^	0.026	−0.25	0.068	−0.17	0.218	−0.29	0.036	−0.20	0.150	−0.14	0.327
Relative β (13–30 Hz) power		0.06	0.679	0.23	0.092	−0.12	0.394	0.027	0.847	0.15	0.280	−0.15	0.290
θ/β ratio		−0.01	0.916	0.02	0.889	0.17	0.219	0.02	0.892	0.05	0.732	0.19	0.177
**Left parietal (P_3_)**													
Relative θ (4–7 Hz) power		0.23	0.096	0.30^#^	0.024	0.27^#^	0.046	0.19	0.172	0.28^#^	0.042	0.25	0.076
Relative α (7–13 Hz) power		−0.35^#^	0.010	−0.38^#^	0.005	−0.37^#^	0.005	−0.30^#^	0.024	−0.33^#^	0.014	−0.33^#^	0.016
Relative β (13–30 Hz) power		0.12	0.395	0.35^#^	0.010	0.17	0.225	0.10	0.492	0.28^#^	0.042	0.10	0.438
θ/β ratio		0.11	0.427	0.04	0.772	0.14	0.321	0.08	0.556	0.06	0.663	0.14	0.321
**Right parietal (P_4_)**													
Relative θ (4–7 Hz) power		0.14	0.325	0.25	0.066	0.22	0.120	0.10	0.487	0.22	0.107	0.18	0.206
Relative α (7–13 Hz) power		−0.13	0.348	−0.25	0.070	−0.23	0.095	−0.08	0.561	−0.19	0.181	−0.17	0.228
Relative β (13–30 Hz) power		−0.11	0.437	0.22	0.110	0.07	0.602	−0.13	0.361	0.14	0.320	0.00	0.991
θ/β ratio		0.20	0.145	0.04	0.767	0.18	0.208	0.18	0.190	0.07	0.631	0.17	0.211

		**Physical neglect**						**Emotional neglect**					
		**Eyes open**		**Eyes closed**		**Go/no-go task**		**Eyes open**		**Eyes closed**		**Go/no-go task**	
		**Spearman**	***p*-Value**	**Spearman**	***p*-Value**	**Spearman**	***p*-Value**	**Spearman**	***p*-Value**	**Spearman**	***p*-Value**	**Spearman**	***p*-Value**

**Left frontal (F_3_)**													
Relative θ (4–7 Hz) power		0.07	0.605	0.21	0.132	0.14	0.317	0.11	0.417	0.22	0.107	0.19	0.176
Relative α (7–13 Hz) power		−0.16	0.250	−0.25	0.074	−0.11	0.444	−0.20	0.142	−0.28^#^	0.038	−0.19	0.171
Relative β (13–30 Hz) power		−0.02	0.860	0.10	0.467	−0.12	0.396	0.02	0.905	0.17	0.237	−0.14	0.326
θ/β ratio		0.11	0.415	0.07	0.619	0.13	0.357	0.04	0.802	0.05	0.747	0.16	0.243
**Right frontal (F_4_)**													
Relative θ (4–7 Hz) power		0.07	0.629	0.17	0.232	0.19	0.181	0.14	0.332	0.13	0.341	0.16	0.249
Relative α (7–13 Hz) power		−0.22	0.108	−0.24	0.088	−0.15	0.272	−0.20	0.148	−0.15	0.298	−0.08	0.581
Relative β (13–30 Hz) power		−0.01	0.958	0.19	0.181	−0.11	0.418	−0.03	0.818	0.11	0.440	−0.18	0.196
θ/β ratio		0.02	0.868	0.04	0.751	0.16	0.266	0.06	0.644	0.03	0.841	0.15	0.268
**Left parietal (P_3_)**
Relative θ (4–7 Hz) power		0.20	0.152	0.30^#^	0.028	0.24	0.082	0.27	0.052	0.30^#^	0.028	0.28^#^	0.036
Relative α (7–13 Hz) power		−0.29^#^	0.031	−0.34	0.013	−0.34^#^	0.011	−0.36^#^	0.008	−0.40^#^	0.003	−0.37^#^	0.006
Relative β (13–30 Hz) power		0.14	0.316	0.33^#^	0.014	0.15	0.287	0.08	0.551	0.35^#^	0.009	0.12	0.410
θ/β ratio		0.08	0.563	0.05	0.747	0.10	0.483	0.17	0.212	0.01	0.968	0.16	0.249
**Right parietal (P_4_)**
Relative θ (4–7 Hz) power		0.14	0.329	0.27	0.051	0.19	0.176	0.17	0.218	0.25	0.067	0.22	0.107
Relative α (7–13 Hz) power		−0.13	0.344	−0.23	0.098	−0.26	0.061	−0.15	0.291	−0.26	0.059	−0.21	0.127
Relative β (13–30 Hz) power		−0.04	0.751	0.21	0.125	0.06	0.668	−0.13	0.361	0.21	0.138	0.01	0.971
θ/β ratio		0.18	0.206	0.09	0.522	0.15	0.290	0.25	0.067	0.03	0.829	0.19	0.171

		**Emotional abuse**						**Denial**					
		**Eyes open**		**Eyes closed**		**Go/no-go task**		**Eyes open**		**Eyes closed**		**Go/no-go task**	
		**Spearman**	***p*-Value**	**Spearman**	***p*-Value**	**Spearman**	***p*-Value**	**Spearman**	***p*-Value**	**Spearman**	***p*-Value**	**Spearman**	***p*-Value**

**Left frontal (F_3_)**													
Relative θ (4–7 Hz) power		0.07	0.626	0.10	0.455	0.24	0.079	−0.27^#^	0.047	−0.24	0.081	−0.22	0.110
Relative α (7–13 Hz) power		−0.14	0.327	−0.14	0.312	−0.21	0.130	0.27^#^	0.047	0.28^#^	0.036	0.21	0.134
Relative β (13–30 Hz) power		−0.01	0.932	0.11	0.434	−0.16	0.264	0.11	0.426	−0.24	0.078	0.15	0.283
θ/β ratio		0.05	0.733	0.05	0.710	0.23	0.105	−0.02	0.908	−0.20	0.155	−0.19	0.182
**Right frontal (F_4_)**													
Relative θ (4–7 Hz) power		0.15	0.280	0.12	0.411	0.32^#^	0.019	−0.15	0.275	−0.15	0.285	−0.14	0.307
Relative α (7–13 Hz) power		−0.27^#^	0.047	−0.11	0.454	−0.21	0.133	0.16	0.245	0.21	0.134	0.21	0.132
Relative β (13–30 Hz) power		−0.07	0.612	0.07	0.612	−0.26	0.062	0.06	0.670	−0.24	0.083	0.04	0.789
θ/β ratio		0.09	0.511	0.11	0.436	0.33^#^	0.014	−0.08	0.551	0.05	0.742	−0.08	0.564
**Left parietal (P_3_)**													
Relative θ (4–7 Hz) power		0.23	0.092	0.19	0.180	0.30^#^	0.025	−0.27	0.052	−0.20	0.151	−0.27^#^	0.044
Relative α (7–13 Hz) power		−0.20	0.150	−0.19	0.166	−0.29^#^	0.032	0.26	0.065	0.25	0.076	0.29^#^	0.032
Relative β (13–30 Hz) power		−0.01	0.928	0.18	0.189	−0.01	0.960	−0.04	0.794	−0.27	0.050	−0.12	0.407
θ/β ratio		0.18	0.203	0.11	0.444	0.25	0.069	−0.23	0.098	0.00	0.993	−0.17	0.222
**Right parietal (P_4_)**													
Relative θ (4–7 Hz) power		0.11	0.439	0.12	0.387	0.24	0.086	−0.24	0.077	−0.20	0.151	−0.29^#^	0.034
Relative α (7–13 Hz) power		0.00	0.987	−0.07	0.604	−0.14	0.328	0.20	0.146	0.29^#^	0.033	0.30^#^	0.024
Relative β (13–30 Hz) power		−0.19	0.166	0.07	0.602	−0.07	0.642	0.05	0.712	−0.29^#^	0.032	−0.13	0.351
θ/β ratio		0.19	0.182	0.07	0.625	0.25	0.068	−0.22	0.116	0.04	0.754	−0.17	0.223

		**Physical abuse**						**Sexual abuse**					
		**Eyes open**		**Eyes closed**		**Go/no-go task**		**Eyes open**		**Eyes closed**		**Go/no-go task**	
		**Spearman**	***p*-Value**	**Spearman**	***p*-Value**	**Spearman**	***p*-Value**	**Spearman**	***p*-Value**	**Spearman**	***p*-Value**	**Spearman**	***p*-Value**

**Left frontal (F_3_)**
Relative θ (4–7 Hz) power		0.02	0.904	0.07	0.634	0.01	0.936	0.05	0.709	0.15	0.297	−0.08	0.553
Relative α (7–13 Hz) power		−0.08	0.555	−0.09	0.543	−0.08	0.593	−0.04	0.797	−0.17	0.226	0.03	0.841
Relative β (13–30 Hz) power		−0.01	0.954	0.00	0.978	0.08	0.564	−0.04	0.795	−0.06	0.668	0.09	0.544
θ/β ratio		0.04	0.750	0.02	0.905	−0.04	0.761	0.16	0.255	0.05	0.712	−0.11	0.427
**Right frontal (F_4_)**													
Relative θ (4–7 Hz) power		0.01	0.940	0.15	0.290	−0.03	0.841	0.12	0.395	0.12	0.411	0.00	0.980
Relative α (7–13 Hz) power		−0.18	0.209	−0.17	0.224	−0.21	0.129	−0.01	0.939	−0.10	0.466	0.05	0.733
Relative β (13–30 Hz) power		0.04	0.796	0.10	0.462	0.17	0.214	−0.14	0.334	−0.06	0.664	−0.04	0.766
θ/β ratio		−0.03	0.855	0.02	0.881	−0.11	0.437	0.13	0.337	0.14	0.309	0.05	0.724
**Left parietal (P_3_)**													
Relative θ (4–7 Hz) power		−0.16	0.258	−0.06	0.652	−0.10	0.488	0.19	0.184	0.14	0.303	0.04	0.772
Relative α (7–13 Hz) power		0.16	0.245	0.12	0.412	0.08	0.582	−0.13	0.336	−0.11	0.451	−0.07	0.626
Relative β (13–30 Hz) power		−0.19	0.183	−0.20	0.156	−0.05	0.742	−0.09	0.531	−0.03	0.820	0.12	0.391
θ/β ratio		0.00	0.977	0.08	0.545	−0.05	0.735	0.15	0.280	0.19	0.170	0.00	0.983
**Right parietal (P_4_)**													
Relative θ (4–7 Hz) power		−0.06	0.645	−0.01	0.930	−0.03	0.809	0.21	0.135	0.17	0.214	0.05	0.745
Relative α (7–13 Hz) power		0.14	0.316	0.08	0.555	0.04	0.781	−0.18	0.209	−0.12	0.376	−0.15	0.299
Relative β (13–30 Hz) power		−0.22	0.120	−0.18	0.200	−0.06	0.695	−0.07	0.638	−0.02	0.901	0.10	0.484
θ/β ratio		0.09	0.540	0.10	0.465	0.02	0.913	0.19	0.182	0.23	0.092	−0.01	0.965

*Correlations between overall ratings and ratings on subscales of the Childhood Trauma Questionnaire and relative brain frequency activity during the stages of the testing session (significant values are indicated as follows, ^#^*p* < 0.05 (with Bonferroni correction applied *p* < 0.00065, *n* = 53)*.

These findings prompted the question as to whether these cortical correlates were associated with a specific form of childhood trauma.

The ratings for neglect (physical and emotional) were significantly negatively correlated with left parietal (P_3_) α band power during all stages of the testing session. In addition, ratings of physical and emotional neglect correlated significantly with left parietal (P_3_) θ and β band power during REC. These results indicate that neglect (physical and emotional) was the main contributor to the association of trauma scores with left parietal electrode findings (Table 1).

Ratings of emotional neglect were significantly negatively correlated with left frontal (F_3_) α band power during REC, similar to the overall childhood trauma ratings. Denial scale ratings correlated with left frontal (F_3_) α band power during REO and REC (Table 1).

Emotional abuse ratings were significantly negatively correlated with left parietal (P_3_) α band power and significantly positively correlated with left parietal (P_3_) θ band power during the GNG. These correlations were reversed for denial ratings; denial ratings were significantly positively correlated with left parietal (P_3_) α band power and significantly negatively correlated with left parietal (P_3_) θ band power during the GNG. Emotional abuse ratings were significantly correlated with right frontal (F_4_) α band power. Emotional abuse ratings were significantly negatively correlated during REO with right frontal (F_4_) θ band power and the θ/β ratio during the GNG (Table 1).

Denial scale ratings were significantly negatively correlated with left frontal (F_3_) θ band power during REO, and with α band power during REO and REC. Denial scale ratings significantly correlated with right parietal (P_3_) activity. During the GNG, denial scale ratings significantly correlated with P_3_ α band power and negatively with θ band power (Table 1).

No cortical correlates were observed for ratings of abuse, either physical or sexual during the various stages of the testing session (Table 1).

#### Cortical physiological measures (EEG) during the various conditions of the GNG task

Within the cognitive task (GNG), there were conditions that required behavioral inhibition and/or delayed stimulus presentation. Relative θ and α band power recorded at the left frontal (F_3_) electrode were different during the various conditions of the GNG [first go condition, first no-go condition, go 3500 condition, second no-go condition, and second go condition, F_3_θ χ^2^_(4,53)_ = 19.21, F_3_α χ^2^_(4.53)_ = 27.4, *p* < 0.001]. Relative α and β band power for right frontal (F_4_) and both parietal (P_3_ and P_4_) electrodes showed differences during the various conditions of the GNG [F_4_α χ^2^_(4.53)_ = 17.38, F_4_β χ^2^_(4,53)_ = 13.79, P_3_α χ^2^_(4,53)_ = 30.08, P_3_β χ^2^_(4,53)_ = 31.01, P_4_α χ^2^_(4,53)_ = 30.36, P_4_β χ^2^_(4,53)_ = 29.10, *p* < 0.001].

**Figure 3:**
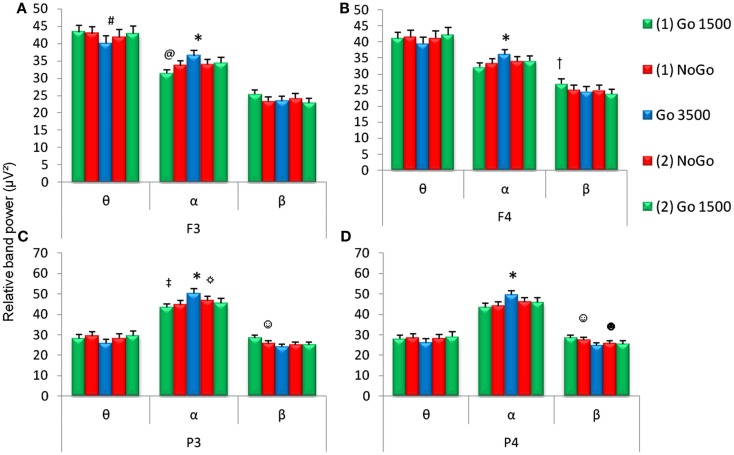
**Relative EEG band power during the five conditions of the GNG task (first go condition, first no-go, go 3500 condition, second go condition, and second no-go conditions)**. Relative frequencies reported include theta (θ, 4–7 Hz), alpha (α, 7–14 Hz), and beta (β, 15–30 Hz) for frontal (F_3_ and F_4_) and parietal (P_3_ and P_4_) electrodes. *For all **(A–D)** α band power was higher during the go 3500 condition compared to first go condition, first no-go, and second no-go condition. **(A)** ^#^F_3_ θ band power was lower during the go 3500 condition compared to the first go condition and first no-go condition and ^@^F_3_ α band power was lower during the first go 1500 condition compared to the second no-go and second go condition. **(B)** ^†^F_4_ β band power was higher during the first go condition compared to the second no-go and second go condition). ^‡^P_3_ α band power was lower during the first go condition compared to the second no-go condition. ^☼^P_3_ α band power was lower during the second go condition compared to the go 3500 condition. ^☺^Parietal **(C,D)** β band power was higher during the first no-go condition compared to the go 3500 condition. **(D)** ^☻^P_4_ β band power was lower during the second go condition compared to the first no-go condition (*p* < 0.0071, *n* = 53, mean ± SEM).

**Figure 4:**
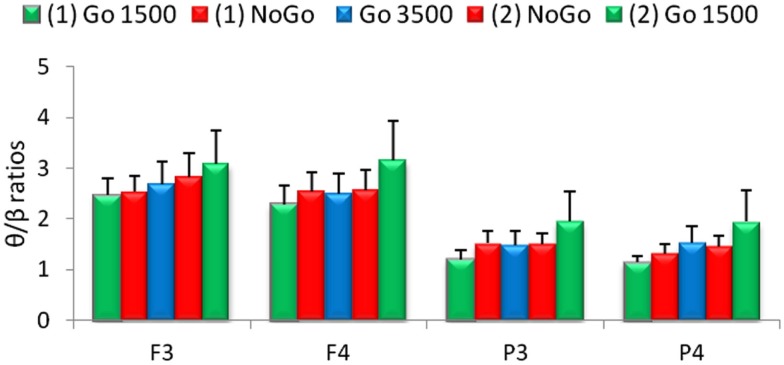
**Relative theta/beta (θ/β) ratios for frontal (F_3_ and F_4_) and parietal (P_3_ and P_4_) electrodes for the five conditions of the GNG task**. No significant differences were found between the various conditions (*n* = 53, mean ± SEM).

Relative α band power was significantly increased during the go 3500 condition compared to the first go condition, first no-go condition, and the second no-go condition for frontal (F_3_ and F_4_) and parietal electrodes (P_3_ and P_4_, Figure 3). Left frontal (F3) relative θ band power significantly decreased during the go 3500 condition compared to the first go condition and first no-go condition. Left frontal (F3) relative α band power was significantly lower during the first go condition compared to the second go condition and second no-go condition (Figure 3). Right frontal (F_4_) relative β band power was significantly greater during the first go condition compared to the second go condition and second no-go condition. Left parietal (P_3_) relative α band power was significantly lower during the first go condition compared to the second no-go condition. Left parietal (P_3_) α band power was significantly lower during the second go condition when compared to the go 3500 condition. Parietal (P_3_ and P_4_) relative β band power was significantly greater during the first no-go condition compared to the go 3500 condition. Right parietal (P_4_) relative β band power was significantly lower during the second go condition compared to the first no-go condition (Figure 3). No significant differences were found in θ/β ratios during the different conditions of the GNG testing session (Figure 4).

**Figure 5:**
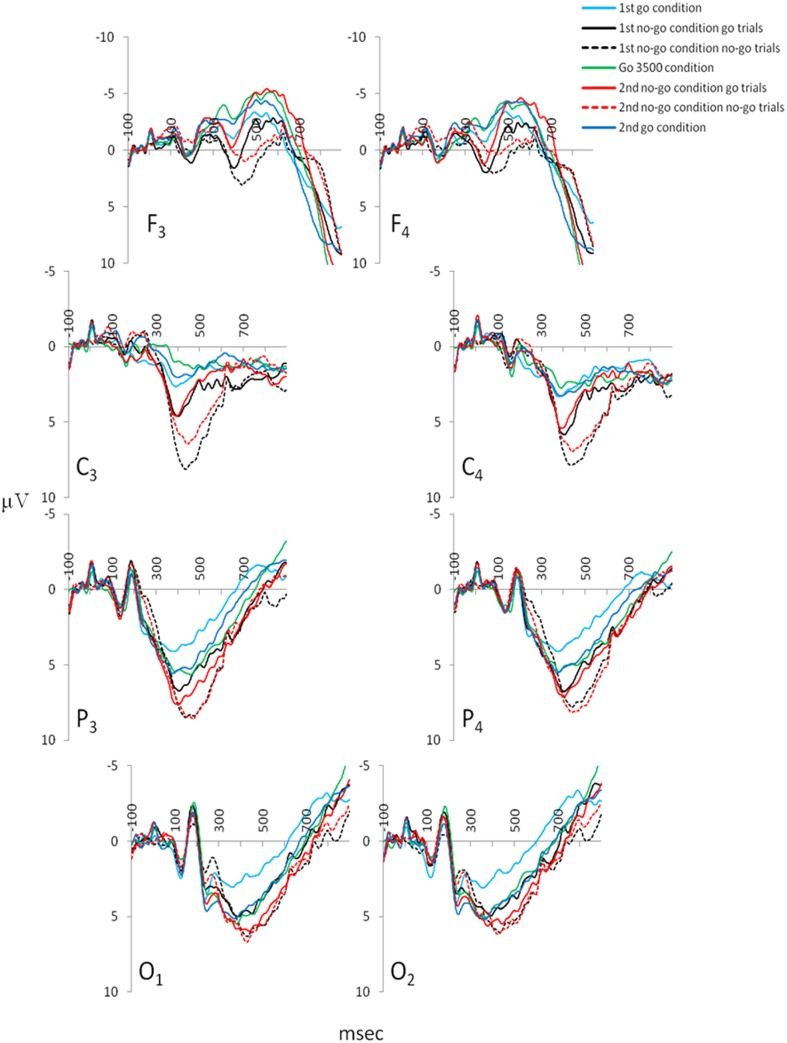
**Grand mean ERPs extracted for the various conditions of the GNG: frontal electrodes (F_3_ and F_4_), central electrodes (C3 and C4), parietal electrodes (P_3_ and P_4_) from which the P300 amplitudes and latencies were extracted, and occipital electrodes (O1 and O2; *n*_grand mean average_ = 53)**.

The P300 amplitudes and latencies extracted over the parietal cortices (P_3_ and P_4_) revealed several differences in the GNG task [P_3_ P300 amplitude χ^2^_(6,53)_ = 33.18, P_3_ P300 latency χ^2^_(6,53)_ = 37.64, P_4_ P300 amplitude χ^2^_(6,53)_ = 27.32, P_4_ P300 latency χ^2^_(6,53)_ = 32.71, *p* < 0.001]. Figure 5 depicts the ERPs of frontal (F_3_ and F_4_), central (C_3_ and C_4_), parietal (P_3_ and P_4_), and occipital (O_1_ and O_2_) electrodes in the GNG task. For both parietal electrodes (P_3_ and P_4_) P300 amplitude during the first go condition was significantly smaller than during the second no-go condition go trials and the first and second no-go condition no-go trials (Table 2). The P300 amplitude during the go 3500 condition was significantly smaller than during the second no-go condition no-go trials. The P300 amplitude during the second go condition was significantly smaller than the first and second no-go condition no-go trials (Table 2). The parietal (P_3_ and P_4_) P300 latency during the first go condition was significantly shorter than during the first no-go condition go trials and the first and second no-go conditions no-go trials. Parietal (P_3_ and P_4_) P300 latency during the go 3500 condition and the second go condition was significantly shorter than the first and second no-go conditions no-go trials (Table 2).

**Table 2:** **Parietal P300 amplitudes (a) and latencies (b) observed during the conditions of the GNG**.

	Left parietal (P_3_)		Right parietal (P_4_)	
	Mean	SEM	Mean	SEM
**(a)**				
**P300 AMPLITUDE (μV)**				
First go condition	6.92*	0.58	6.55*	0.55
First no-go condition go trials	9.29	0.84	9.14	0.78
First no-go condition no-go trials	11.03	0.90	9.33	0.72
Go 3500 condition	8.16**	0.57	7.89**	0.57
Second no-go condition go trials	9.70	0.77	8.99	0.79
Second no-go condition no-go trials	10.88	0.66	10.32	0.81
Second go condition	8.11***	0.49	7.64***	0.50
**(b)**				
**P300 LATENCY (ms)**				
First go condition	391.23^#^	16.70	372.19^#^	15.35
First no-go condition go trials	440.04	14.06	433.23	13.30
First no-go condition no-go trials	471.00	9.21	457.19	10.42
Go 3500 condition	420.88^##^	13.91	412.23^##^	12.96
Second no-go condition go trials	436.00	12.25	421.31	13.77
Second no-go condition no-go trials	467.69	11.52	442.00	11.27
Second go condition	402.69^##^	11.82	395.69^##^	12.33

*P300 amplitude during the first go condition was significantly smaller compared to no-go trials (first and second no-go conditions) and the go trials during the second no-go condition. **P300 amplitude during go 3500 condition was significantly smaller than the no-go trials of the second no-go condition. ***P300 amplitude during second go condition was significantly smaller than the no-go trials (first and second conditions). ^#^P300 latency was significantly shorter during the first go condition than the no-go trials (first and second no-go conditions) and go trials of the first no-go condition. ^##^P300 latency was significantly shorter than during the no-go trials (first and second no-go conditions; *p* < 0.05, *n* = 53).

#### Associations between childhood trauma and cortical physiological measures (EEG) during the various conditions of the GNG task

Three significant associations were found between ratings on the Childhood Trauma Questionnaire (excluding the denial scale) and cortical measures of arousal during the various conditions of the GNG (first go condition, first no-go condition, go 3500 condition, second no-go condition, and second go condition; Table 3). Overall scores on the Childhood Trauma questionnaire were correlated with left parietal (F_3_) β band power during the go 3500 condition and left parietal (F_3_) θ band power during the second go condition. In addition, right parietal (P_4_) P300 amplitude during the first no-go condition no-go trials correlated with overall scores on the Childhood Trauma Questionnaire. Inclusion of the denial scale resulted in a single significant correlation between left parietal (P_3_) P300 latency during the first no-go condition no-go trials and childhood trauma.

**Table 3:** **Correlations between the participants’ overall ratings on the Childhood Trauma Questionnaire and cortical physiological EEG measures during the various conditions of the GNG**.

	CTQ score without denial scale										CTQ score with denial scale									
	(1) Go 1500		(1) No-go 1500		Go 3500		(2) No-go 1500		(2) Go 1500		(1) Go 1500		(1) No-go 1500		Go 3500		(2) No-go 1500		(2) Go 1500	
	Spearman	*p*-Value	Spearman	*p*-Value	Spearman	*p*-Value	Spearman	*p*-Value	Spearman	*p*-Value	Spearman	*p*-Value	Spearman	*p*-Value	Spearman	*p*-Value	Spearman	*p*-Value	Spearman	*p*-Value
**Left frontal (F_3_)|**																				
Relative θ (4–7 Hz) power	−0.08	0.580	0.08	0.604	−0.01	0.927	0.01	0.970	0.16	0.287	−0.10	0.514	0.04	0.812	0.00	0.993	−0.01	0.971	0.12	0.422
Relative α (7–13 Hz) power	0.08	0.622	−0.04	0.814	−0.03	0.824	0.07	0.626	−0.16	0.299	0.08	0.583	0.04	0.814	−0.02	0.904	0.09	0.568	−0.07	0.653
Relative β (13–30 Hz) power	−0.01	0.965	−0.06	0.674	0.10	0.506	−0.07	0.624	0.00	0.980	0.00	0.999	−0.05	0.724	0.07	0.661	−0.08	0.610	−0.03	0.841
θ/β ratio	−0.01	0.964	0.07	0.630	−0.04	0.814	0.04	0.809	0.15	0.324	−0.01	0.941	0.06	0.713	−0.01	0.950	0.03	0.823	0.14	0.348
**Right frontal (F_4_)**																				
Relative θ (4–7 Hz) power	0.04	0.771	0.11	0.460	0.05	0.765	0.05	0.734	0.23	0.136	0.07	0.645	0.08	0.592	0.08	0.591	0.06	0.694	0.20	0.190
Relative α (7–13 Hz) power	0.09	0.562	−0.05	0.766	−0.08	0.593	0.07	0.656	−0.13	0.390	0.08	0.610	0.02	0.912	−0.07	0.628	0.09	0.558	−0.06	0.699
Relative β (13–30 Hz) power	−0.10	0.509	−0.13	0.405	0.07	0.645	−0.09	0.551	−0.05	0.760	−0.12	0.429	−0.15	0.334	0.02	0.916	−0.13	0.408	−0.08	0.581
θ/β ratio	0.07	0.657	0.14	0.374	0.00	0.978	0.10	0.524	0.20	0.189	0.10	0.533	0.14	0.366	0.06	0.697	0.12	0.436	0.21	0.174
**Left parietal (P_3_)**																				
Relative θ (4–7 Hz) power	0.04	0.772	0.15	0.341	0.15	0.339	0.16	0.291	0.31^#^	0.038	0.02	0.894	0.08	0.580	0.14	0.349	0.17	0.256	0.25	0.093
Relative α (7–13 Hz)power	−0.11	0.483	−0.21	0.172	−0.28	0.065	−0.26	0.083	−0.22	0.139	−0.08	0.582	−0.15	0.339	−0.26	0.088	−0.27	0.074	−0.19	0.216
Relative β (13–30 Hz) power	0.02	0.897	0.06	0.698	0.31^#^	0.036	0.10	0.527	−0.05	0.750	−0.03	0.845	0.04	0.784	0.23	0.121	0.04	0.802	−0.04	0.779
θ/β ratio	0.07	0.644	0.12	0.435	0.01	0.924	0.09	0.566	0.28	0.060	0.06	0.708	0.07	0.646	0.05	0.767	0.10	0.515	0.24	0.112
**Right parietal (P_4_)**																				
Relative θ (4–7 Hz) power	0.07	0.651	0.10	0.496	0.10	0.510	0.04	0.804	0.20	0.190	0.05	0.722	0.05	0.724	0.08	0.600	0.01	0.931	0.14	0.358
Relative α (7–13 Hz) power	0.00	0.988	−0.04	0.812	−0.11	0.471	0.02	0.887	−0.09	0.569	0.05	0.726	0.03	0.832	−0.07	0.652	0.06	0.674	−0.03	0.826
Relative β (13–30 Hz) power	−0.09	0.538	0.00	0.986	0.18	0.235	−0.09	0.562	−0.13	0.378	−0.16	0.283	−0.04	0.770	0.08	0.617	−0.17	0.278	−0.16	0.308
θ/β ratio	0.17	0.272	0.13	0.393	0.07	0.640	0.14	0.345	0.25	0.096	0.17	0.260	0.10	0.501	0.10	0.528	0.15	0.341	0.22	0.146
**P300 amplitude**																				
Left parietal (P_3_)	−0.09	0.569	0.10	0.526	0.21	0.158	−0.02	0.888	0.06	0.712	−0.06	0.695	0.16	0.282	0.16	0.286	0.00	0.982	0.05	0.765
Left parietal (P_3_) for no-go trials			0.15	0.329			0.14	0.348					0.18	0.246			0.07	0.664		
Right parietal (P_4_)	0.06	0.703	0.10	0.494	0.26	0.083	0.07	0.667	0.09	0.547	−0.04	0.772	−0.09	0.556	−0.08	0.616	0.03	0.852	0.14	0.355
Right parietal (P_4_) for no-go trials			0.29^#^	0.048			0.18	0.228					0.12	0.446			−0.01	0.936		
**P300 latency**																				
Left parietal (P_3_)	−0.06	0.675	0.00	0.979	−0.17	0.262	0.05	0.768	0.18	0.241	0.11	0.475	0.19	0.201	0.24	0.105	0.10	0.493	0.09	0.554
Left parietal (P_3_) for no-go trials			0.13	0.399			0.09	0.565					0.33^#^	0.024			0.14	0.351		
Right parietal (P_4_)	−0.06	0.704	0.10	0.514	−0.02	0.875	0.08	0.613	0.02	0.915	−0.09	0.561	−0.01	0.967	0.06	0.706	0.08	0.622	−0.04	0.783
Right parietal (P_4_) for no-go trials			0.15	0.314			0.13	0.413					0.13	0.404			0.02	0.896		

*Correlations between subscales of the Childhood Trauma Questionnaire and relative brain frequency activity and P300 amplitudes and latencies during the conditions of the go/no-go task [significant values are indicated as follows, #*p* < 0.05 (with Bonferroni correction applied *p* < 0.00071), *n* = 53]*.

Few significant associations were found between total CTQ scores and cortical activity during the GNG tasks conditions, associations with CTQ subscales were examined during the GNG tasks conditions (first go condition, first no-go condition, go 3500 condition, second no-go condition, and second go condition).

Significant correlations were found between subscales of neglect (physical and emotional) and cortical activity during the GNG, as left parietal (P_3_) α band power was negatively correlated during the go 3500 condition and second no-go condition and θ band power was positively correlated during the second no-go condition. In addition, emotional neglect significantly correlated with left parietal (P_3_) θ band power during the second go condition and correlated significantly negatively with α band power during the first no-go condition and second go condition (Table 4).

**Table 4:** **Correlations between the participants’ ratings on subscales of the Childhood Trauma Questionnaire and cortical physiological EEG measures during the various conditions of the GNG**.

	(1) Go 1500		(1) No-go 1500		Go 3500		(2) No-go 1500		(2) Go 1500		(1) Go 1500		(1) No-go 1500		Go 3500		(2) No-go 1500		(2) Go 1500	
	Spearman	*p*-Value	Spearman	*p*-Value	Spearman	*p*-Value	Spearman	*p*-Value	Spearman	*p*-Value	Spearman	*p*-Value	Spearman	*p*-Value	Spearman	*p*-Value	Spearman	*p*-Value	Spearman	*p*-Value
		
	Physical neglect										Emotional neglect									
**Left frontal (F_3_)**																				
Relative θ (4–7 Hz) activity	−0.04	0.762	0.06	0.691	0.02	0.889	0.08	0.576	0.13	0.366	−0.04	0.779	0.13	0.371	0.08	0.573	0.12	0.399	0.21	0.125
Relative α (7–13 Hz) activity	0.10	0.472	−0.01	0.946	−0.04	0.787	−0.14	0.332	−0.07	0.607	0.02	0.894	−0.13	0.344	−0.13	0.363	−0.12	0.412	−0.25	0.071
Relative β (13–30 Hz) activity	−0.06	0.646	−0.09	0.530	−0.01	0.918	−0.08	0.550	−0.09	0.506	−0.05	0.735	−0.07	0.615	−0.03	0.808	−0.14	0.318	−0.04	0.799
θ/β ratio	0.04	0.771	0.09	0.503	0.04	0.763	0.08	0.578	0.16	0.266	0.02	0.863	0.11	0.446	0.08	0.554	0.15	0.294	0.16	0.245
**Right frontal (F_4_)**																				
Relative θ (4–7 HZ) activity	0.00	0.987	0.11	0.415	0.09	0.499	0.15	0.270	0.23	0.102	0.04	0.762	0.10	0.475	0.08	0.575	0.10	0.463	0.24	0.085
Relative α (7–13 Hz) activity	0.01	0.934	−0.06	0.695	−0.11	0.447	−0.14	0.303	−0.12	0.393	0.08	0.561	−0.02	0.911	−0.08	0.565	−0.03	0.849	−0.17	0.227
Relative β (13–30 Hz) activity	0.04	0.774	−0.08	0.580	0.01	0.961	−0.10	0.464	−0.11	0.416	−0.12	0.374	−0.15	0.289	−0.09	0.514	−0.17	0.210	−0.12	0.379
θ/β ratio	−0.02	0.902	0.12	0.400	0.06	0.664	0.14	0.328	0.20	0.152	0.08	0.546	0.15	0.298	0.07	0.629	0.17	0.234	0.20	0.151
**Left parietal (P_3_)**																				
Relative θ (4–7 Hz) activity	0.03	0.828	0.13	0.358	0.13	0.353	0.28^#^	0.041	0.25	0.071	0.12	0.390	0.21	0.122	0.21	0.132	0.29^#^	0.034	0.32^#^	0.017
Relative α (7–13 Hz)activity	−0.17	0.220	−0.24	0.090	−0.28^#^	0.037	−0.37^#^	0.005	−0.27	0.052	−0.19	0.179	−0.28^#^	0.038	−0.32^#^	0.016	−0.36^#^	0.007	−0.30^#^	0.029
Relative β (13–30 Hz) activity	0.20	0.149	0.17	0.235	0.27	0.052	0.05	0.697	0.09	0.505	0.09	0.528	0.09	0.540	0.24	0.086	0.03	0.847	−0.03	0.844
θ/β ratio	−0.03	0.828	0.03	0.852	0.01	0.948	0.13	0.338	0.15	0.280	0.11	0.423	0.16	0.261	0.06	0.667	0.17	0.222	0.25	0.070
**Right parietal (P_4_)**																				
Relative θ (4–7 Hz) activity	−0.01	0.943	0.04	0.762	0.12	0.396	0.18	0.199	0.18	0.204	0.12	0.382	0.16	0.255	0.17	0.226	0.22	0.109	0.24	0.084
Relative α (7–13 Hz) activity	0.01	0.961	−0.10	0.481	−0.19	0.181	−0.20	0.143	−0.18	0.204	−0.07	0.610	−0.13	0.336	−0.17	0.237	−0.16	0.267	−0.18	0.189
Relative β (13–30 Hz) activity	−0.02	0.862	0.09	0.528	0.18	0.204	−0.07	0.611	−0.04	0.790	−0.07	0.615	0.04	0.801	0.10	0.463	−0.13	0.359	−0.11	0.453
θ/β ratio	0.06	0.667	0.02	0.906	0.09	0.541	0.17	0.224	0.16	0.246	0.16	0.265	0.14	0.318	0.14	0.321	0.24	0.089	0.24	0.086
**P300 amplitude**																				
Left parietal (P_3_)	−0.03	0.859	−0.01	0.937	0.12	0.395	0.05	0.733	−0.07	0.608	−0.05	0.721	−0.18	0.192	0.11	0.447	0.09	0.510	0.10	0.496
Left parietal (P_3_) for no-go trials			0.13	0.352			0.00	0.975					0.16	0.260			0.21	0.136		
Right parietal (P_4_)	−0.03	0.818	0.22	0.122	−0.14	0.335	0.15	0.295	0.03	0.818	−0.01	0.971	0.22	0.109	0.06	0.671	0.14	0.323	0.14	0.306
Right parietal (P_4_) for no-go trials			0.14	0.337			0.04	0.774					0.23	0.108			0.13	0.354		
**P300 latency**																				
Left parietal (P_3_)	0.04	0.782	−0.07	0.621	0.14	0.340	−0.02	0.878	−0.04	0.792	−0.10	0.487	−0.20	0.153	0.17	0.224	0.04	0.790	0.09	0.515
Left parietal (P_3_) for no-go trials			0.00	0.985			−0.01	0.932					−0.09	0.518			0.17	0.218		
Right parietal (P_4_)	0.08	0.585	−0.04	0.761	−0.03	0.856	−0.06	0.692	0.12	0.389	0.04	0.790	0.02	0.902	−0.05	0.708	0.02	0.912	0.17	0.227
Right parietal (P_4_) for no-go trials			0.05	0.723			0.00	0.993					0.11	0.438			0.07	0.598		

	**Emotional abuse**										**Denial**									

**Left frontal (F_3_)**																				
Relative θ (4–7 Hz) activity	0.13	0.340	0.22	0.109	0.18	0.201	0.23	0.096	0.20	0.144	−0.20	0.159	−0.31^#^	0.021	−0.12	0.380	−0.20	0.160	−0.31^#^	0.024
Relative α (7–13 Hz) activity	−0.05	0.747	−0.04	0.792	−0.17	0.234	−0.12	0.399	−0.10	0.478	0.14	0.304	0.31^#^	0.023	0.14	0.327	0.14	0.314	0.34^#^	0.011
Relative β (13–30 Hz) activity	−0.16	0.262	−0.27	0.050	−0.05	0.747	−0.26	0.062	−0.17	0.236	0.16	0.246	0.18	0.191	0.02	0.859	0.16	0.266	0.08	0.579
θ/β ratio	0.17	0.236	0.27^#^	0.043	0.15	0.292	0.25	0.067	0.23	0.102	−0.20	0.151	−0.24	0.083	−0.09	0.511	−0.19	0.181	−0.22	0.113
**Right frontal (F_4_)**																				
Relative θ (4–7 Hz) activity	0.20	0.148	0.25	0.070	0.23	0.093	0.31^#^	0.022	0.26	0.057	−0.13	0.358	−0.27	0.051	−0.06	0.695	−0.14	0.318	−0.28^#^	0.043
Relative α (7–13 Hz) activity	−0.02	0.910	−0.06	0.674	−0.22	0.122	−0.13	0.368	−0.09	0.531	0.11	0.426	0.30^#^	0.030	0.19	0.177	0.18	0.198	0.28	0.040
Relative β (13–30 Hz) activity	−0.28^#^	0.042	−0.34^#^	0.013	−0.10	0.492	−0.31^#^	0.020	−0.21	0.126	0.08	0.553	0.08	0.584	−0.06	0.666	0.07	0.642	0.08	0.588
θ/β ratio	0.25	0.071	0.34^#^	0.012	0.19	0.163	0.35^#^	0.009	0.28^#^	0.039	−0.09	0.499	−0.19	0.184	0.02	0.896	−0.10	0.464	−0.19	0.167
**Left parietal (P_3_)**																				
Relative θ (4–7 Hz) activity	0.12	0.373	0.23	0.099	0.22	0.112	0.23	0.095	0.22	0.110	−0.19	0.168	0.36^#^	0.007	−0.18	0.190	−0.24	0.081	−0.34^#^	0.014
Relative α (7–13 Hz)activity	−0.03	0.847	−0.15	0.292	−0.23	0.101	−0.23	0.098	−0.14	0.326	0.16	0.246	0.32^#^	0.019	0.22	0.112	0.20	0.142	0.26	0.063
Relative β (13–30 Hz) activity	−0.10	0.490	−0.07	0.629	0.11	0.449	−0.07	0.639	−0.13	0.359	−0.09	0.510	−0.02	0.906	−0.24	0.088	−0.10	0.479	0.09	0.506
θ/β ratio	0.19	0.166	0.24	0.084	0.17	0.235	0.23	0.099	0.23	0.091	−0.16	0.257	−0.30^#^	0.026	−0.03	0.850	−0.16	0.242	−0.29^#^	0.035
**Right parietal (P_4_)**																				
Relative θ (4–7 Hz) activity	0.12	0.373	0.16	0.248	0.16	0.252	0.19	0.183	0.13	0.355	−0.15	0.293	−0.30^#^	0.029	−0.22	0.119	−0.30^#^	0.028	−0.32^#^	0.020
Relative α (7–13 Hz) activity	0.06	0.675	0.01	0.932	−0.09	0.517	−0.06	0.659	−0.03	0.858	0.19	0.169	0.30^#^	0.031	0.25	0.073	0.26	0.061	0.30^#^	0.030
Relative β (13–30 Hz) activity	−0.22	0.122	−0.11	0.419	0.04	0.779	−0.21	0.131	−0.19	0.181	−0.08	0.576	−0.09	0.531	−0.26	0.063	−0.11	0.431	0.03	0.845
θ/β ratio	0.27	0.052	0.24	0.078	0.18	0.196	0.29^#^	0.035	0.20	0.161	−0.12	0.410	−0.23	0.093	−0.08	0.579	−0.20	0.141	−0.25	0.070
**P300 amplitude**																				
Left parietal (P_3_)	0.00	0.980	0.26	0.067	0.22	0.112	0.11	0.448	0.07	0.621	0.06	0.672	0.24	0.082	−0.05	0.709	−0.14	0.311	−0.07	0.604
Left parietal (P_3_) for no-go trials			0.20	0.153			0.14	0.313					0.01	0.935			−0.18	0.205		
Right parietal (P_4_)	−0.12	0.398	0.04	0.757	−0.06	0.659	−0.15	0.281	0.02	0.881	−0.05	0.736	−0.24	0.086	0.15	0.279	−0.15	0.291	−0.07	0.622
Right parietal (P_4_) for no-go trials			0.16	0.270			0.06	0.669					−0.05	0.703			−0.17	0.230		
**P300 latency**																				
Left parietal (P_3_)	−0.12	0.415	0.24	0.090	0.16	0.254	0.01	0.951	0.06	0.675	0.05	0.718	0.20	0.157	−0.16	0.254	−0.11	0.424	−0.09	0.539
Left parietal (P_3_) for no-go trials			0.18	0.207			0.16	0.265					0.07	0.628			−0.16	0.247		
Right parietal (P_4_)	−0.08	0.554	−0.05	0.702	−0.18	0.189	−0.05	0.741	0.14	0.329	0.06	0.671	−0.12	0.401	0.19	0.186	−0.16	0.262	−0.07	0.623
Right parietal (P_4_) for no-go trials			0.20	0.146			0.14	0.324					−0.05	0.743			−0.10	0.462		

	**Physical abuse**										**Sexual abuse**									

**Left frontal (F_3_)**																				
Relative θ (4–7 Hz) activity	−0.09	0.534	−0.07	0.600	0.04	0.752	0.03	0.840	0.02	0.901	−0.15	0.300	−0.15	0.290	−0.05	0.738	−0.15	0.299	−0.04	0.795
Relative α (7–13 Hz) activity	0.04	0.799	−0.02	0.865	−0.11	0.426	−0.04	0.793	−0.06	0.693	0.12	0.411	0.06	0.649	−0.04	0.778	0.14	0.305	0.00	0.979
Relative β (13–30 Hz) activity	0.17	0.220	0.15	0.289	0.12	0.379	0.06	0.668	0.14	0.331	0.07	0.630	0.05	0.710	0.15	0.293	0.07	0.594	0.05	0.706
θ/β ratio	−0.12	0.388	−0.11	0.418	−0.03	0.822	−0.04	0.753	−0.06	0.647	−0.11	0.415	−0.12	0.384	−0.11	0.429	−0.14	0.319	−0.05	0.696
**Right frontal (F_4_)**																				
Relative θ (4–7 Hz) activity	−0.14	0.325	−0.12	0.386	0.01	0.961	−0.04	0.771	−0.05	0.742	−0.01	0.966	−0.12	0.386	0.01	0.963	−0.03	0.806	0.00	1.000
Relative α (7–13 Hz) activity	−0.04	0.794	−0.07	0.609	−0.22	0.106	−0.10	0.457	−0.09	0.513	0.08	0.546	0.10	0.485	0.02	0.885	0.10	0.476	0.01	0.929
Relative β (13–30 Hz) activity	0.19	0.173	0.18	0.207	0.18	0.191	0.15	0.296	0.19	0.163	−0.03	0.856	−0.02	0.905	0.06	0.687	−0.03	0.814	0.03	0.825
θ/β ratio	−0.17	0.216	−0.18	0.202	−0.07	0.630	−0.11	0.441	−0.14	0.328	0.02	0.907	−0.05	0.697	0.00	0.984	0.01	0.939	−0.02	0.863
**Left parietal (P_3_)**																				
Relative θ (4–7 Hz) activity	−0.28^#^	0.040	−0.12	0.374	−0.06	0.651	−0.12	0.374	−0.06	0.644	−0.06	0.673	−0.10	0.466	0.11	0.438	0.01	0.960	0.07	0.601
Relative α (7–13 Hz)activity	0.29^#^	0.032	0.20	0.152	0.04	0.795	0.13	0.348	0.10	0.473	−0.04	0.770	0.00	0.975	−0.13	0.338	−0.13	0.342	−0.13	0.367
Relative β (13–30 Hz) activity	−0.04	0.791	0.00	0.995	0.00	0.987	−0.12	0.389	−0.10	0.483	0.12	0.405	0.12	0.394	0.22	0.120	0.07	0.621	0.15	0.269
θ/β ratio	−0.17	0.223	−0.06	0.682	0.01	0.961	0.00	0.987	−0.02	0.878	−0.12	0.402	−0.11	0.418	0.01	0.962	−0.04	0.784	−0.03	0.821
**Right parietal (P_4_)**																				
Relative θ (4–7 Hz) activity	−0.23	0.102	−0.08	0.592	−0.01	0.926	−0.11	0.418	0.01	0.926	0.01	0.948	−0.02	0.867	0.11	0.424	0.00	0.986	0.12	0.373
Relative α (7–13 Hz) activity	0.28^#^	0.046	0.16	0.239	−0.01	0.950	0.14	0.327	0.03	0.850	−0.07	0.608	−0.05	0.703	−0.19	0.181	−0.10	0.487	−0.14	0.319
Relative β (13–30 Hz) activity	−0.06	0.688	−0.06	0.653	0.02	0.887	−0.13	0.369	−0.09	0.507	0.09	0.517	0.08	0.579	0.21	0.140	0.04	0.752	0.09	0.503
θ/β ratio	−0.14	0.324	0.01	0.971	0.01	0.924	−0.01	0.941	0.05	0.744	−0.05	0.721	−0.06	0.670	0.03	0.856	−0.02	0.876	0.01	0.956
**P300 amplitude**																				
Left parietal (P_3_)	0.27	0.056	0.13	0.353	0.26	0.061	−0.03	0.834	0.19	0.172	0.14	0.309	0.31^#^	0.026	0.26	0.067	0.03	0.839	0.05	0.718
Left parietal (P_3_) for no-go trials			0.37^#^	0.006			0.25	0.068					0.20	0.153			0.18	0.193		
Right parietal (P_4_)	−0.16	0.271	−0.08	0.570	0.07	0.603	0.05	0.747	0.04	0.764	−0.14	0.323	0.06	0.687	0.05	0.706	−0.18	0.195	−0.19	0.183
Right parietal (P_4_) for no-go trials			0.07	0.617			0.39^#^	0.004					0.00	0.994			0.06	0.665		
**P300 latency**																				
Left parietal (P_3_)	−0.03	0.827	−0.03	0.841	0.12	0.397	−0.11	0.432	0.07	0.616	0.01	0.956	0.22	0.120	0.13	0.347	0.06	0.697	0.00	0.977
Left parietal (P_3_) for no-go trials			0.13	0.373			0.15	0.284					0.30^#^	0.033			0.20	0.164		
Right parietal (P_4_)	−0.01	0.932	−0.11	0.449	0.01	0.970	0.13	0.373	0.16	0.246	−0.05	0.718	0.03	0.808	0.05	0.703	−0.25	0.070	0.02	0.882
Right parietal (P_4_) for no-go trials			−0.04	0.770			0.30^#^	0.031					−0.02	0.861			0.33^#^	0.018		

*Correlations between subscales of the Childhood Trauma Questionnaire and relative brain frequency activity and P300 amplitudes and latencies during the conditions of the go/no-go task [significant values are indicated as follows, ^#^*p* < 0.05 (with Bonferroni correction applied *p* < 0.00071), *n* = 53]*.

Ratings of emotional abuse correlated significantly and negatively with β band power during the first go and the two no-go conditions, and correlated significantly and positively with right frontal (F_4_) θ/β ratios during the first and second no-go conditions and the second go condition (Table 4).

Denial scale scores were significantly and positively correlated with θ band power and negatively correlated with α, for frontal (F_3_ and F_4_) and parietal (P_3_ and P_4_) electrodes during the first no-go condition and the second go condition. In addition, ratings on the denial scale showed a significant and negative correlation with the left parietal (P_3_) θ/β ratio during the first no-go condition and second go condition (Table 4).

Significant EEG correlates of physical and sexual childhood abuse were found only during the GNG. Physical abuse correlated negatively with θ band power and positively with α band power in the parietal cortices (P_3_ and P_4_) during the first go condition, while left parietal (P_3_) θ band power during the first go condition negatively correlated with ratings of physical abuse (Table 4).

Physical and sexual childhood abuse were the only subscales of the Childhood Trauma Questionnaire that correlated significantly with the P300 amplitude and latency. Physical abuse correlated positively with left parietal (P_3_) P300 amplitude during the no-go trials of the first no-go condition and right parietal (P_4_) P300 amplitude and latency during the no-go trials of the second no-go condition. Sexual abuse correlated positively with left parietal (P_3_) P300 amplitude during the go trials of the first no-go condition, with left parietal (P_3_) P300 latency during the no-go trials of the the first no-go condition, and right parietal (P_4_) P300 latency during the no-go trials of the second no-go condition (Table 4).

### Peripheral physiological measures during the testing session

#### Skin conductance (responses and duration) and heart rate

Skin conductance responses and their duration were different during the various stages of the testing session (REO, REC, and GNG). Differences were found in the number of skin conductance responses [χ^2^_(2,53)_ = 75.01, *p* < 0.0001] and duration of responses [χ^2^_(2,53)_ = 12.35, *p* < 0.001] during the different stages of the testing session. The number of skin conductance responses was fewer during REO (3.5, 0.6) and REC (2.3, 0.4) than during the GNG task (19.4, 2.7), which would be expected, as the duration of the task was longer than the periods of rest (each 2 min long; *p* < 0.0001). Fewer responses were made during REC than REO (*p* < 0.01). The duration of skin conductance responses was shorter during REO (2225 ± 405 ms) than during REC (3359 ± 351 ms) and the GNG (3444 ± 257 ms, all *p* < 0.0001). Heart rate during the various stages of the testing session (REO, REC, and GNG) was different [χ^2^_(2,53)_ = 45.7, *p* = 0.0001]. Heart rate was higher during the GNG (78 ± 2 bpm) than during REO (74 ± 1.8 bpm) and REC (75 ± 1.9 bpm, all *p* < 0.0001). Heart rate during REC was higher than during REO (*p* < 0.001).

The number of skin conductance responses differed for the various conditions of the GNG task [χ^2^_(2,53)_ = 75.01, *p* = 0.0001]. There were significantly fewer skin conductance responses during the go 3500 condition (2.1 ± 0.3) compared to the first go condition (3.7 ± 0.5), first no-go condition (4.3 ± 0.5), and second no-go condition (3.6 ± 0.5). Significantly fewer skin conductance responses were made during the second go condition (2.8 ± 0.5) than during the first no-go condition. However, the duration of skin conductance responses during the various conditions of the GNG was not different (first go condition = 2929 ± 283 ms, first no-go condition = 4174 ± 625 ms, go 3500 condition = 2383 ± 361 ms, second no-go = 3009 ± 313 ms, and second go condition = 2647 ± 344 ms).

#### Response times during the GNG task

Response times differed during the various conditions of the GNG [χ^2^_(5,53)_ = 93.29, *p* < 0.0001], shorter response times were found during the first go condition (383 ± 6.2 ms) compared to the first no-go (430 ± 3.2 ms), go 3500 condition (424 ± 4.3 ms), second no-go (437 ± 2.7 ms), and the second go condition (403 ± 4.8 ms). The second go condition also showed significantly shorter response times compared to the no-go conditions and the go 3500 condition. In the first no-go condition there were 1.6 ± 1.5 errors made, in the second no-go condition there were 1.1 ± 1.1 errors made.

#### Salivary cortisol

**Figure 6:**
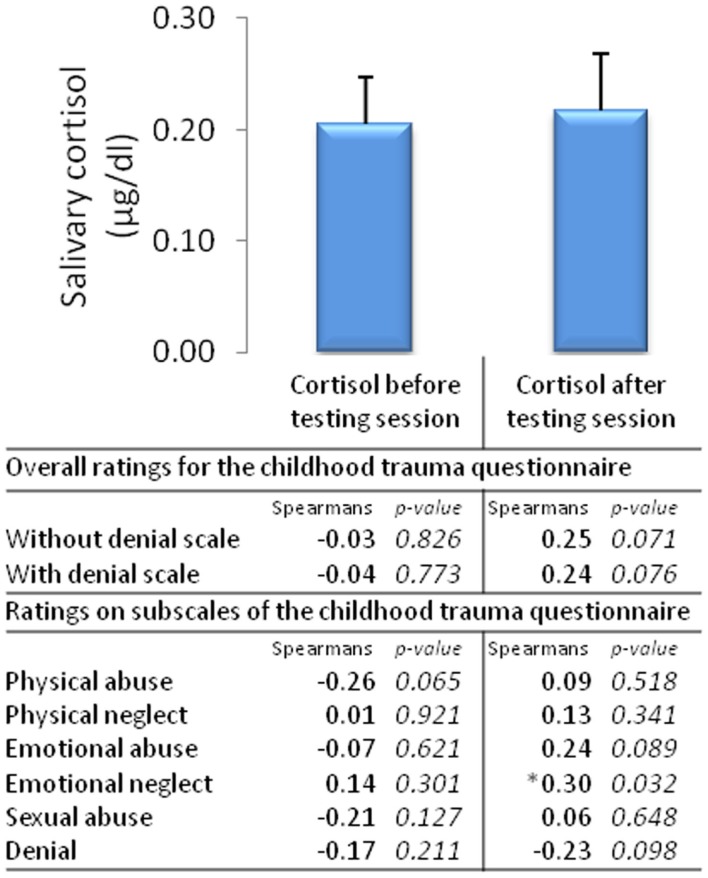
**Salivary cortisol before and after the testing session and correlates of overall ratings and ratings on subscales of the Childhood Trauma Questionnaire (*n* = 53, mean ± SEM)**.

Salivary cortisol was taken at the start of the testing session and at the end of the testing session, with no difference in cortisol detected between the start and the end of the testing session [χ^2^_(1,53)_ = 1.92, *p* = 0.17, Figure 6].

#### Associations between childhood trauma and peripheral physiological measures

The only peripheral physiological measure of arousal that significantly correlated with childhood trauma were cortisol levels taken after the testing session, emotional abuse was positively correlated with cortisol after the testing session (Figure 6). No other peripheral measures (e.g., skin conductance, heart rate, and response times) correlated significantly with childhood trauma ratings.

## Discussion

Childhood trauma ratings were significantly associated with several patterns of cortical arousal, and with one measure of peripheral arousal. Our main findings were: (1) physical and emotional neglect correlated with decreased left parietal tonic α band power during resting conditions and during the GNG task; (2) emotional abuse correlated with decreased right frontal α band power during rest increased θ band power during the GNG task, and cortisol at the end of the testing session; (3) physical and sexual abuse correlated with delayed P300 latency and enhanced P300 amplitude during the no-go conditions of the GNG task; and (4) the denial scale correlated with a decrease in θ and increase in α band power during the no-go conditions of the GNG task.

### EEG associations with childhood neglect

Physical and emotional neglect during childhood were associated with increased cortical arousal in adulthood, with decreased α band power over the left parietal cortex. Decreased α band activity has previously been associated with increased arousal, attention, and increased mental load (Ray and Cole, 1985; Klimesch, 1999; Fink et al., 2005), while decreased left hemisphere α activity has been associated with increased approach behavior (Sutton, 1997) and increased novelty-seeking behavior (Glass and Butler, 1977; Klimesch et al., 2001). These findings are consistent with previous work suggesting that physical and emotional childhood neglect may result in increased arousal and increased approach/novelty-seeking behaviors in adulthood.

### EEG associations with childhood emotional abuse

Emotional abuse in childhood was associated with increased arousal in adulthood during conditions requiring behavioral inhibition, as seen by increased frontal θ and decreased right α band power. Emotional abuse was the only subscale to show significant associations with right hemispheric activity. Within several of the GNG task conditions, β band power decreased and the θ/β ratio increased. The pattern of significant correlations with the right frontal hemisphere was activity dependent; that is, activation of behavioral inhibition circuitry was required (Sutton, 1997; Barry et al., 2009). Emotional abuse was correlated with salivary cortisol, the only peripheral arousal association. An infant study indicates that activation of behavioral inhibition is associated with increased plasma cortisol (Buss et al., 2003). We suggest that childhood emotional abuse is associated with increased cortical arousal when the individual activates behavioral inhibition networks.

### EEG associations with childhood abuse

Physical and sexual abuse correlated with delayed P300 latency and enhanced P300 amplitude during the no-go conditions of the GNG task. Thus these early childhood traumas are associated with slowed information processing and enhanced cortical updating in adulthood. This may reflect more deliberate information processing that would serve to prevent a negative outcome of an impulsive response.

### EEG associations with under-reporting of childhood trauma

The denial scale correlated with a decrease in θ and an increase in α band power during the no-go conditions of the GNG task. The denial scale ratings showed decreased θ band power and increased α band power in a random pattern during various stages of the testing session. However, during the GNG task this pattern was clearly evident globally; that is, bilaterally and fronto-posteriorly. The two conditions of the GNG that displayed a strong pattern of activation were the first behavioral inhibition condition and the condition after the last behavioral inhibition condition. This suggests that the denial scale scores were associated with activation of behavioral inhibition circuitry followed by disengagement of this circuitry. As θ band power has been shown to increase with increased mental effort (Smit et al., 2004a,b; Howells et al., 2010), it is possible that denial of childhood trauma is associated with increased mental effort during activation and deactivation of inhibitory neural circuitry.

## Limitations of the Present Study

The present study has several limitations. The sample was one of “convenience” as it comprised volunteers from the researchers’ home institution, and may not generalize to the broader community. The EEG provided an average of multiple surface field potentials, and therefore could not be localized to any particular area of the brain. Skin conductance levels, a measure of tonic peripheral arousal, were not reported: only skin conductance responses, a measure of phasic peripheral arousal, were reported. The design of the present study was cross-sectional; to determine causal relationships a longitudinal research design should be carried out, and caution is therefore required in interpreting these data. Furthermore, an individual’s report of childhood trauma may be subject to recall bias, so that we are uncertain whether we are studying objective exposure to trauma or merely perceptions of such trauma. However retest reliability of the Childhood Trauma Questionnaire has been established (Bernstein et al., 1994, 2003), which suggests that recall is not subject to changes in arousal. Finally, in this preliminary hypothesis-generating paper we did not correct for multiple testing, and some findings may reflect false positives.

## Conclusion

The present data suggest that childhood trauma is associated with enduring psychobiological changes that are evident in adulthood. More specifically, childhood trauma is associated with increased cortical arousal and shows particular patterns of cortical activity depending on the form of childhood trauma experienced. Given that these associations were found in a non-clinical population, it would be particularly relevant to determine whether they also are found in psychiatric disorders characterized by deficits in arousal.

## Conflict of Interest Statement

The authors declare that the research was conducted in the absence of any commercial or financial relationships that could be construed as a potential conflict of interest.

## Abbreviations

EEG, electroencephalography; ERPs, event-related potentials; GNG, go/no-go task; REC, resting eyes closed; REO, resting eyes open.
